# Secondary Syphilitic Nephritis

**Published:** 1909-09-25

**Authors:** 


### SECONDARY SYPHILITIC NEPHRITIS.

It is unfortunate that so much stress is usually laid upon the occurrence of œdema in acute nephritis that most medical students and many medical men find it difficult to believe that acute nephritis can be present unless there is puffiness of the eyelids, swelling of the ankles, or other evidence of œdema. It is well enough proved, however, that a virulent and fatal acute nephritis may run its entire course without any œdema at all. The fact has been confirmed by autopsy repeatedly, after scarlet fever for example. If the urine were not tested systematically in these cases, the occurrence of acute nephritis might be entirely missed. It is so missed every day simply because urines are not tested regularly during every fever. Hence it is that many a case of chronic nephritis arises without assignable cause when really it is the natural and direct outcome of an acute nephritis that passed unrecognised years before.

Nor is it only after scarlet fever that this non-œdematous form of acute nephritis occurs. It is by no means uncommon in connection with pneumococcal diseases, especially lobar pneumonia complicated with empyema. It is also to be seen in cases of secondary syphilis. The albuminuria of secondary syphilis is sometimes regarded merely as a symptomatic albuminuria, chiefly on account of the absence of general œdema; but frequently, if the urine is examined microscopically, plenty of renal epithelial cells and tube-casts indicative of actual nephritis will be found. It sometimes happens there is general œdema as well, so that the nephritic nature of the albuminuria becomes obvious to all. That the nephritis in such a case is actually syphilitic has been proved by the recovery of the *Treponema pallidum* of Schaudinn from the urine. This has been observed by Dreyer, Toepel, Hirschberg, MacLennan, Barth, and Michaux. The following is a case in point:—

A young woman of twenty-eight came under observation two months after contracting syphilis. She was covered with an abundant and typical roseola, together with buccal and vulval mucous plaques. She also exhibited a generalised anasarca that had developed suddenly the day before in her face, and had thence spread in four and twenty hours all over her body and limbs. The heart was natural; there was no ascites and no pleural effusion. The urine was very scanty—less than five ounces in the twenty-four hours. It was high-coloured, and contained fifteen parts of albumin per thousand, together with a few red blood corpuscles and many fatty and granular tube-casts. Some of the deposit was specially stained, and after some search typical examples of the Treponema pallidum were found, and their nature was confirmed by a skilled bacteriologist. No other bacteria were seen.

The patient was at once placed on the intensive treatment of daily intra-muscular injections of benzoate of mercury—two centigrams a day. The diet was strictly limited to milk, and absolute rest in bed was enjoined. At the end of two days the anasarca was getting less and the quantity of urine passed was increasing. In ten days' time the œdema had entirely gone, and the albuminuria ceased a few days after that. The intra-muscular injections were changed for oral administration after the twelfth.

It is interesting to note that whatever may be the case when non-syphilitic nephritis occurs in a syphilitic subject, in the above case, in which the nephritis itself was syphilitic, mercurial treatment was well borne.

